# Detection and prevalence of antimicrobial resistance genes in multidrug-resistant and extensively drug-resistant *Staphylococcus* and *Streptococcus* species isolated from raw buffalo milk in subclinical mastitis

**DOI:** 10.1371/journal.pone.0324920

**Published:** 2025-06-17

**Authors:** Md. Shahidur Rahman Chowdhury, Hemayet Hossain, Md. Bashir Uddin, Md. Matiur Rahman, Ferdaus Mohd Altaf Hossain, Md. Rafiqul Islam, M. Nazmul Hoque, Md. Masudur Rahman, Md. Mukter Hossain, Md. Mahfujur Rahman

**Affiliations:** 1 Department of Medicine, Sylhet Agricultural University, Sylhet, Bangladesh; 2 Department of Anatomy and Histology, Sylhet Agricultural University, Sylhet, Bangladesh; 3 Department of Dairy Science, Sylhet Agricultural University, Sylhet, Bangladesh; 4 Department of Gynecology, Obstetrics and Reproductive Health, Gazipur Agricultural University, Gazipur, Bangladesh; 5 Department of Pathology, Sylhet Agricultural University, Sylhet, Bangladesh; Universidad San Francisco de Quito, ECUADOR

## Abstract

Subclinical mastitis (SCM) poses a significant threat to the global dairy industry, particularly in Bangladesh, where it remains a major constraint in buffalo dairy farming. The rising prevalence of antimicrobial resistant pathogens complicates disease management, resulting reduced milk yield, increased veterinary expenses, compromised animal welfare and potential risk to public health. This study investigated the prevalence and resistance profiles of multidrug-resistant (MDR) and extensively drug-resistant (XDR) *Staphylococcus* and *Streptococcus* species in raw buffalo milk from SCM cases in Bangladesh. A total of 1,540 quarter milk samples from 385 buffaloes were analyzed, revealing SCM prevalence rates of 67.9% (1046/1540; 95% CI: 65.6–70.3) at the quarter level and 80.8% (311/385; 95% CI: 76.5–84.6) at the animal level. Notable regional variations were observed, with Gowainghat showing the highest prevalence (88.1%; 141/160). This study did not identify any biologically plausible risk factors for the occurrence of SCM in buffalo. The Modified Whiteside Test and California Mastitis Test confirmed SCM, with culture and biochemical tests identifying 789 (51.2%) *Staphylococcus* spp. and 424 (27.5%) *Streptococcus* spp. isolates. Polymerase Chain Reaction (PCR) analysis indicated that 72.7% (456/627) of *Staphylococcus* isolates were *Staphylococcus aureus*, while the predominant *Streptococcus* species included *Streptococcus uberis* (32.3%) and *Streptococcus dysgalactiae* (14.9%). Resistance gene detection revealed a high prevalence of antimicrobial resistant genes (ARGs), particularly *aac-3(iv)* and *tetA,* across different buffalo quarters and habitats. Antibiogram profiling demonstrated high susceptibility to tetracycline (80.9; 83.1) and trimethoprim-sulfamethoxazole (87.4; 81.9), while significant resistance was noted against ampicillin (88.8; 87.1) and nalidixic acid (68.1; 62.1). MDR was observed in 76.4% (479/627) of *Staphylococcus* spp. and 67.3% (167/248) of *Streptococcus* spp. isolates, with 10.37% (65/627) and 10.48% (26/248) classified as possible XDR, respectively. These findings explored high antimicrobial resistance level among *Staphylococcus* and *Streptococcus* species in subclinical mastitis, highlighting the need for improved management practices and surveillance to mitigate public health risks posed by contaminated milk.

## Introduction

Buffalo farming plays a crucial role in the livelihoods of rural communities worldwide, contributing significantly to milk and meat production [1]. Despite their adaptability to diverse environmental conditions and resilience to harsh climates, buffaloes remain underutilized compared to cattle in many regions, including Bangladesh. In this context, improving buffalo productivity faces challenges such as subclinical mastitis (SCM), a silent but detrimental disease affecting milk quality, animal health, and farm economics [2]. In Bangladesh, two predominant types of buffalo, the Riverine and Swamp types, are reared, although their breed diversity remains largely unexplored [3]. Riverine buffaloes are primarily found in the coastal regions, while Swamp buffaloes are commonly raised in the marshy haor areas of Sylhet and Kishoreganj [4]. In Sylhet, haor areas such as Kanaighat, Jaintapur, Gowainghat, Balaganj, and Fenchuganj are notable for their marshy landscapes, making them well-suited for Swamp buffalo production [3]. Despite its potential, buffalo farming in Bangladesh is not widely popular. However, initiatives like the Buffalo Development Project aim to promote buffalo farming across the country. In these regions, buffaloes are typically reared under household systems with minimal inputs, often managed as small units through scavenging practices [5,6].

The expansion of buffalo farming in Asia has been significant, adding nearly 13% to worldwide milk production over the past 50 years [7]. Despite these advancements, mastitis remains a major issue, adversely affecting milk production and leading to economic losses, frequent antibiotic use, and compromised animal health [8,9]. It causes an average annual economic loss of $70 per buffalo, with 55% due to intervention costs and 16% to reduced milk yield [10]. In dairy animals, mastitis can be clinical (CM) or subclinical (SCM), with SCM being more prevalent and economically detrimental, occurring 15–40 times more frequently than CM [11]. SCM often goes undetected due to the absence of visible symptoms, contributing to long-term losses in milk production and quality. The etiology of SCM is multifactorial, influenced by microbial factors such as pathogen virulence and load, host factors like immune response and nutritional status, and environmental factors including management practices, milking procedures, and the surrounding ecological setting [12]. In tropical climates, where buffalo farming is prevalent, the risk of SCM is significantly increased. Injuries to the udder or teats, combined with poor milking hygiene and suboptimal management practices, can contribute to a greater susceptibility to infection [13]. Among the pathogens responsible for SCM, environmental pathogen like *Streptococcus uberis*, and contagious agents like *Staphylococcus aureus* and *Streptococcus agalactiae*. *Streptococcus dysgalactiae* can complicate the disease dynamics [14].

*Staphylococcus aureus* is a widespread zoonotic pathogen that affects both humans and animals, contributing to significant health challenges across various sectors [15,16]. Methicillin-resistant strains specially MRSA, leading to higher mortality rates and increased healthcare costs [15]. Studies indicate that it is responsible for approximately one-third of SCM cases, attributed to its production of toxins, virulence determinants, and cell wall adhesion proteins [17,18]. One of the most alarming characteristics of *S. aureus* is its ability to adapt and resist various antibiotics. This is facilitated by the presence of antimicrobial resistance (AMR) genes, which enable the bacteria to resist to various environments and develop resistance to commonly used antibiotics [19]. Over time, this has led to the emergence of multidrug-resistant *S. aureus* (MDR-SA) strains, including MRSA [15,19]. These resistant strains are more difficult to treat and control, further complicating efforts to manage SCM in dairy herds [20]. As MRSA continues to evolve under selective pressures from antibiotic use, some strains have progressed to extensively drug resistance, posing them a serious threat to both animal and public health [21,22].

*Streptococcus* (*Streptococcus agalactiae, Streptococcus dysgalactiae,* and *Streptococcus uberis*) is another major group of bacterial pathogens accounting for 23%–50% of all mastitis cases worldwide [23]. In the past, 90% of mastitis cases were attributed to *Streptococcus agalactiae* [24] and causes higher Somatic Cell Count (SCC) thereby compromising milk quality [25,26]. In recent years, the emergence of multidrug-resistant (MDR) and extensively drug-resistant (XDR) *Streptococcus* and *Staphylococcus* have become a significant public health concern. The increasing prevalence of antimicrobial resistance poses a serious threat in Bangladesh. A study on urinary tract infections (UTIs) reported that 54.2% of isolates exhibited MDR, while 7% were identified as XDR [27]. Similarly, among isolates from pus samples in Bangladesh, 74.02% of *Staphylococcus* spp. displayed MDR, and 66.6% of *Streptococcus* spp. were classified as XDR [28]. In the context of livestock, *Staphylococcus* spp. isolated from subclinical mastitis (SCM) milk samples demonstrated 21.8% MDR, with no evidence of XDR or pan-drug resistance (PDR) [29]. The detection of the *mecA* gene in *Staphylococcus aureus* isolates, classifying 61.1% as MRSA, represents a significant public health threat [29].

These resistant strains exhibit resistance to multiple antibiotics complicating treatment strategies. Additionally, MDR strains can cause more severe infections due to enhanced biofilm formation and increased virulence, making both prevention and management of these infections increasingly difficult [30,31]. Likewise, environmental *Streptococci*, particularly *Streptococcus uberis*, also an important cause of mastitis worldwide and can lead to significant economic detriment [32]. Moreover, a recent study reported that *Streptococcus dysgalactiae* causes mastitis with an average incidence of 55.0% mild, 38.7% intermediate, and 6.3% severe, with high excretion rates compared to other mastitogens [24,33]. The current research explored the prevalence and resistant genes in multidrug-resistant and extensively drug-resistant *S. aureus* and *Streptococcus* spp. in raw milk samples from SCM affected buffaloes.

## Materials and methods

### Ethical consideration

The study received approval from the Institutional Ethics Committee of Sylhet Agricultural University, Sylhet-3100, Bangladesh, under animal use protocol number #AUP2023001.

### Study design, location and Sampling strategy

**Fig 1:**
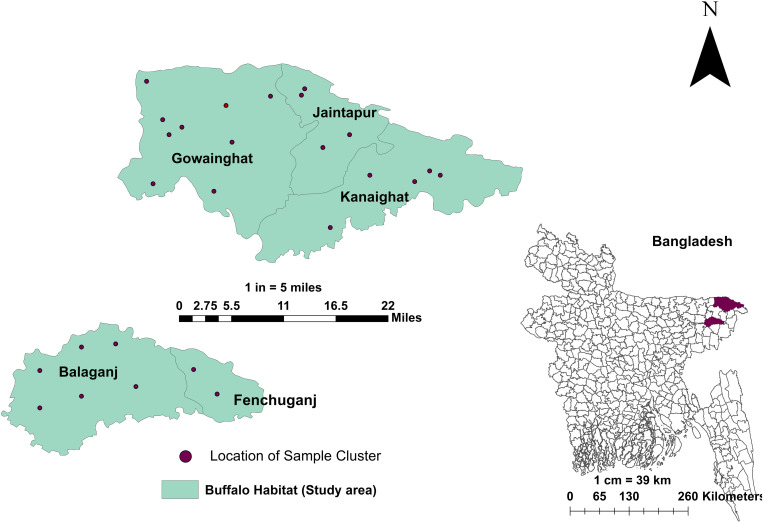
Geographical mapping of study area. The map was created using ArcMap 10.8.


A cross-sectional investigation was conducted in five Upazilas predominantly buffalo populated area in the Sylhet district of Bangladesh, namely Jaintapur, Gowainghat, Kanaighat, Balaganj, and Fenchuganj. These regions are situated within the geographic coordinates of approximately 24°36’ to 25°11’ North latitude and 91°38’ to 92°30’ East longitude, depicted in Fig 1. The study population required to estimate prevalence was calculated using a standard equation [34,35].

$$n=\frac{Z^{2}\times Pexp\times \left(1-Pexp\right)}{d^{2}}$$

Where, n = Desired sample size;

Z = 1.96 for 95% confidence interval;

P_exp_ = 0.5, Expected prevalence (50%);

d = 0.05, Desired absolute precision (5%)

Based on the calculations, milk sample from 384 buffaloes (1536 quarter milk samples) were required to estimate the prevalence of SCM. The study proceeded with the collection of 1540 quarter milk samples from 385 buffaloes to determine both quarter-level and animal-level prevalence. The samples were collected using a random-cluster sampling strategy from February 2023 to June 2024. Within each selected cluster, animals were randomly chosen regardless of their age, parity, or lactation stage.

### Initial screening of SCM

The milk samples were collected aseptically from each quarter of apparently healthy buffaloes, following the guidelines set by the National Mastitis Council (NMC) [36]. The Modified Whiteside test (MWST), outlined by Emon et al. [37] along with the California Mastitis Test (CMT) [38] were utilized as the initial screening methods for detecting subclinical mastitis. After aseptic collection of the milk, the samples were mixed with a CMT reagent in a specified ratio on a CMT paddle. The mixture was then gently swirled, and the reaction was observed for changes in consistency. The grading system ranged from negative (Grade 0), where no change in viscosity occurred and the milk remained liquid, to strong (Grade 3+), which was characterized by a very thick consistency and pronounced gel formation. Intermediate grades, such as slight thickening with no gel formation (Graded as Trace), noticeable thickening with slight coagulation (Grade 1+), and thick consistency with pronounced gel formation (Grade 2+), indicated varying levels of thickening and gel formation/ coagulation of SCM positive milk. These grades were determined based on the formation of gel and/or coagulation of the milk on the CMT paddle (S1 Table). The primarily positive milk samples underwent a pre-enrichment in Trypticase Soya Broth (HiMedia Laboratories Pvt. Ltd., Mumbai, India) at a 1:10 dilution. Subsequent incubation of these cultures was at 37°C for around 24 hours.

### Isolation and identification of pathogens

The isolation and identification of *S. aureus* was performed using Mannitol Salt Agar (Oxoid, UK) following the guidelines and procedures of NMC, USA. The colonies appeared yellow due to mannitol fermentation, typically shiny and smooth, after incubation at 37°C for 18–24 hours [39]. Biochemical tests confirmed the presence of *S. aureus* with positive results of Gram staining, catalase, coagulase, citrate utilization, methyl red (MR), Voges-Proskauer (VP), urease tests and DNase test while negative results were obtained for motility, indole, gas production, and the triple sugar iron test [37].

For the isolation of *Streptococcus* species (*Streptococcus agalactiae, Streptococcus dysgalactiae, Streptococcus uberis*), Modified Edwards Medium Base (Oxoid, UK) was utilized followed by Hassan et al. [23]. After incubation at 37°C for 24–48 hours, small, greyish colonies exhibiting alpha or beta hemolysis were noted following the addition of 5–7% sterile de-fibrinated sheep blood. Confirmation of the *Streptococci* was achieved through biochemical testing, which yielded positive results for gram staining and the triple sugar iron test. In contrast, all other tests, including catalase, coagulase, citrate utilization, motility, indole, gas production, methyl red, and urease, returned negative results [40]. Following these analyses, positive samples were prepared for genomic DNA extraction and polymerase chain reaction (PCR).

### Genomic DNA extraction

The established boiling method was employed to extract genomic DNA from *S. aureus* and various *Streptococcus* spp. as previously outlined by Aldous et al. [41]. The quality and concentration of the extracted DNA were assessed utilizing a NanoDrop 2000c spectrophotometer (Thermo Fisher Scientific Inc., Waltham, MA, USA). The monoplex PCR assays targeted the *tuf, nuc, mecA, aac (3)-iv,* and *sul1* gene. The multiplex PCR assays focused on the *sip, 16S rRNA, pau, tetA*, and *strA* gene. The sequences of the primers used in both the monoplex and multiplex PCR assays are detailed in Table 1.

**Table 1:** The sequences of primer with amplicon sizes, and target genes used for PCR-based identification of specific organisms and resistance genes.

Primer set	Organism	Target gene	Primer sequence (5’– 3’)	PCR type	Product size(bp)	Reference
**1**	Genus Streptococcus	*tuf*	F: CAACTTGACGAAGGTCCTGCA R: TGGGTTGATTGAACCTGGTTTA	Monoplex	**110**	[42]
**2**	*Staphylococcus aureus*	*nuc*	F: GCGATTGATGGTGATACGGTT R: AGCCAAGCCTTGACGAACTAAAGC	Monoplex	**267**	[42]
**3**	Methicillin resistant *Staphylococcus aureus*	*mecA*	F: TGCTATCCACCCTCAAACAGG R: AACGTTGTAACCACCCCAAGA	Monoplex	**286**	[42]
**4**	*Streptococcus* *agalactiae*	*sip*	F: CTATTGACATCGACAATGGCAGC R: GTTACTGTCAGTGTTGTCTCAGGA	Multiplex	**266**	[42]
**5**	*Streptococcus* *dysgalactiae*	*16s rRNA*	F: GGAGTGGAAAATCCACCAT R: CGGTCAGGAGGATGTCAAGAC		**549**	[42]
**6**	*Streptococcus* *uberis*	*pau*	F: TGCTACTCAACCATCAAAGGTTGC R: TAGCAGTCTCAGTAGGATGAGTGA		**439**	[42]
**7**	Gentamicin resistance gene	*aac (3)-iv*	F: AGTTGACCCAGGGCTGTCGC R: GTGTGCTGCTGGTCCACAGC	Monoplex	**333**	[43]
**8**	Sulfonamide resistance gene	*sul1*	F: CGGCGTGGGCTACCTGAACG R: GCCGATCGCGTGAAGTTCCG	Monoplex	**433**	
**9**	Tetracycline resistance gene	*tetA*	F: GGCGGTCTTCTTCATCATGC R: CGGCAGGCAGAGCAAGTAGA	Multiplex	**502**	
**10**	Streptomycin resistance gene	*strA*	F: ATGGTGGACCCTAAAACTCT R: CGTCTAGGATCGAGACAAAG		**893**	

### Evaluation of diagnostic test efficacy

To evaluate the diagnostic performance of the MWST as screening test for SCM, the sensitivity, specificity, positive predictive value (PPV), negative predictive value (NPV), positive likelihood ratio (LR^+^), negative likelihood ratio (LR^-^) and accuracy were calculated. The MWST based results were validated against CMT, which was considered as gold standard screening test for SCM diagnosis. The following formulas were used [44]:


$$Sensitivity\;=\;\frac{True\;positive\;(TP)}{True\;positive\;(TP)+False\;negative\;(FN)}\;\times 100$$

$$Specificity\;=\;\frac{True\;Negative\;(TN)}{True\;Negative\;(TN)+False\;positive\;(FP)}\;\times 100$$

$$Positive\;Predictive\;Value\;(PPV)\;=\;\frac{True\;Positive\;(TP)}{True\;Positive\;(TP)+False\;positive\;(FP)}\;\times 100$$

$$Negative\;Predictive\;Value\;(NPV)\;=\;\frac{True\;Negative\;(TN)}{True\;Negative\;(TN)+False\;Negative\;(FN)}\;\times 100$$

$$Positive\;Likelihood\;Ratio\;(LR+)\;=\;\frac{Sensitivity}{\left(100-Specificity\right)}$$

$$Negative\;Likelihood\;Ratio\;(LR-)\;=\;\frac{\left(100-Sensitivity\right)}{Specificity}$$

$$Accuracy\;=\;\frac{TP+TN}{TP+TN+FP+FN}\;\times 100$$

### Molecular detection of pathogens and ARGs

The molecular detection of *Staphylococcus* and *Streptococcus* species and antibiotic resistance genes was conducted using different PCR techniques. For the detection of the *Streptococcus* genus and *S. aureus*, monoplex PCR targeting the *tuf* gene and the *nuc* gene, respectively, was performed using reagents from SFC Probes Ltd. (Gyeonggi, Korea). Likewise, resistance to gentamicin and sulfonamides was assessed by monoplex PCR amplification of the *aac-3(iv)* and *sul1* genes, respectively. Additionally, a separate monoplex PCR was employed to identify MRSA by targeting the *mecA* gene. Multiplex PCR assays were executed to amplify specific genes for *Streptococcus agalactiae (sip), Streptococcus dysgalactiae (16S rRNA),* and *Streptococcus uberis (pau),* along with *tetA* and *strA* genes for tetracycline and streptomycin resistance, respectively. The compositions of the PCR reaction mixture and amplification conditions for these assays are detailed in S2 Table. All amplified products were verified through gel electrophoresis on a 1.8% or 1.5% low-melting agarose gel, using a 100 bp plus ladder for size verification. The primer sequences for all targeted genes are outlined in Table 1.

### Antimicrobial susceptibility testing

Antimicrobial Susceptibility testing (AST) was executed employing the Kirby-Bauer disk diffusion method on Mueller-Hinton agar (MHA) plate following the guidelines of Clinical and Laboratory Standards Institute (CLSI) [45]. A total of 13 antibiotics spanning 8 distinct groups, including penicillin’s (ampicillin 10 µg, amoxicillin-clavulanic acid 20/10 µg), tetracycline (tetracycline 30 µg), aminoglycosides (gentamicin 10 µg, amikacin 30 µg, streptomycin 10 µg), macrolides (azithromycin 15 µg), quinolones (ciprofloxacin 5 µg, nalidixic acid 30 µg), folate pathway antagonists (trimethoprim-sulfamethoxazole 1.25/23.75 µg), phenicol (chloramphenicol 30 µg), and cephalosporins (cefoxitin 30 µg, ceftriaxone 30 µg). Freshly isolated bacterial colonies were suspended in 5 mL of normal saline to obtain a density of 0.5 McFarland. This suspension was evenly spread on MHA plates, and incubated at 35–37°C for 18–24 hours. After incubation, the inhibition zone diameters were measured in millimeters (mm) and compared to CLSI breakpoints. Each assay was conducted in triplicate to ensure accuracy and reproducibility [46].

### MAR, MDR and XDR

The Multiple Antibiotic Resistance (MAR) index was ascertained following the protocol delineated by Naser et al. [34], utilizing the equation: MAR = (Number of antibiotics exhibiting resistance by an isolate)/ (Total number of antibiotics subjected to testing). MAR index values spanned from 0 to 1, where values closer to zero indicated higher susceptibility, while those nearing 1 signified pronounced resistance. A MAR index equal to or exceeding 0.20 was indicative of a high-risk reservoir of bacterial contamination or a notable level of resistance. In addition, non-susceptibility to at least one agent in three classes of antibiotics or three antimicrobial categories is designated as MDR [47] while non-susceptibility to at least one agent in all but 2 or fewer antimicrobial categories is termed as XDR [48].

### Statistical analysis, geo-spatial mapping and plot

Excel spreadsheets were employed for the meticulous compilation, organization, and structuring of the accumulated data. A multivariate logistic regression analysis was conducted to identify potential risk factors, following the methodology described by Farabi et al. [29] with some modifications. All relevant independent variables associated with the prevalence of SCM were first subjected to univariate analysis using the Chi-square (χ²) test. Variables with a *p*-value ≤ 0.20 in the univariate analysis were initially considered for multivariate logistic regression. However, due to the limited number of significant variables, a more inclusive threshold of *p* ≤ 0.80 was used to select variables for the multivariate model.

A multivariate logistic regression analysis was then conducted to identify potential risk factors associated with SCM. The farm ID was included as a random effect variable to account for clustering at the farm level. A backward stepwise regression approach was used to refine the final model. Collinearity among independent variables was assessed using Fisher’s exact test, and variables with a *p*-value ≤ 0.05 were considered collinear and excluded from the model.

To assess the model’s validity, the Hosmer-Lemeshow test was used to evaluate its goodness-of-fit. The predictive accuracy of the final model was determined using the receiver operating characteristic (ROC) curve. Statistical significance was set at *p* < 0.05. All data analyses were performed using SPSS version 26 (SPSS Inc., Chicago, IL, USA). The results were reported following established multivariate regression reporting guidelines [49]. ArcMap 10.8 (ArcMap 10.8, Esri, USA) was used to generate the study area mapping, with a shapefile sourced from (www.diva-gis.org). The creation of dot map effectively visualizes the sample cluster of buffalo habitat in Sylhet, Bangladesh.

## Results

### Animal and quarter level prevalence

The overall prevalence of SCM based on CMT was 67.9% (1046/1540; 95% CI: 65.6–70.3) at the quarter level and 80.8% (311/385; 95% CI: 76.5–84.6) at the animal level (Table 2).

**Table 2:** Univariable (Chi-square) analysis of the prevalence of SCM at the animal level (AL) and quarter level (QL) in swamp buffaloes across different geographic regions of Sylhet. The prevalence of SCM was expressed as percent (%) with 95% CI value with level of significance.

Attributes (Buffalo Habitat)	Level of Measures (N)	x (%)	95% CI	Level of Significance
Kanaighat	AL (70)	42 (60.0)	47.6-71.5	NS
	QL (280)	163 (58.2)	52.2-64.1	
Jaintapur	AL (80)	72 (90.0)	81.2-95.6	*
	QL (320)	234 (73.1)	67.9-77.9	
Gowainghat	AL (40)	40 (100)	91.2-100^a^	*
	QL (160)	141 (88.1)	82.1-92.7	
Balaganj	AL (105)	91 (86.7)	78.6-92.5	*
	QL (420)	268 (63.8)	59.0-68.4	
Fenchuganj	AL (90)	66 (73.3)	62.9-82.1	*
	QL (360)	240 (66.7)	61.5-71.5	
**Overall**	AL (385)	311 (80.8)	76.5-84.6	**
	QL (1540)	1046 (67.9)	65.5-70.3	

^a^Indicates one-sided 97.5% Confidence interval; Chi-square test; x: number of positive isolates, N: number of samples tested, CI: Confidence interval; AL: Animal level; QL: Quarter level; NS: Non-significant, *P < 0.05, **P < 0.01

The research covered multiple buffalo habitats, showing that Gowainghat had the highest SCM rates, with 100% (40/40) at the selected animal level and 88.1% (141/160) at the quarter level followed by Jaintapur, 90% (72/80) at animal level and 73.1% (234/320) at quarter level. Kanaighat recorded the lowest prevalence, with 60% (42/70) at animal level and 58.2% (163/280) at quarter level. Among the CMT-positive samples (N = 1046), 37.48% were graded as Trace. Additionally, 20.46% of the samples received a grade of 1 + , while 28.01% were graded as 2 + , and 14.05% were graded as 3 + .

### Factors contributing SCM

The prevalence of SCM in buffaloes influenced by several factors (Table 3). Early lactation (1–2 months of lactation) buffaloes exhibited the highest prevalence of SCM at 85.7% (60/70), while mid-lactation (3–6 months) buffaloes showed the lowest prevalence at 74.8% (104/139). Buffaloes with two or fewer parities displayed the prevalence of 83.4% (126/151), compared to those with three or more parities. Milking frequency also affected the rates, with twice-daily milking showing a rate of 79.4% (189/238) and once-daily milking showing a rate of 82.9% (122/147). Intriguingly, buffaloes without a previous history of mastitis had 78.9% (150/190) positive for SCM. Other pivotal factors encompassed body condition score (81.2% for 3 or higher), muddy flooring (83.3%; 195/239), pendulous udders (86.1%; 192/252), funnel-shaped teats (87.3%; 191/241), and high stocking density (86.9%; 163/208).

**Table 3:** Multivariable mixed-effects logistic regression analysis of subclinical mastitis (defined as CMT score ≥ 1) regressed against the animal level risk factors of 385 buffalo in 248 farms expressed as odds ratio (OR), 95% confidence interval (95% CI), and *P* value.

Variable	Category	x/N	% (95% CI)	β-Co-efficient (OR)	Std. error	Z-value	*P*-value
**Intercept**				**0.57**	**0.58**	**0.99**	**0.32**
**Lactation stage**	>7 months	104/139	83.5 (77.2-88.7)	−0.02	2.17	−0.009	0.99
	3-6 months	147/176	74.8 (66.8-81.8)	0.04	2.19	0.02	0.98
	1-2 months	60/70	85.7 (75.3-92.9)	Ref.			
**Parity**	≤2	126/151	83.4 (76.5-88.9)	0.08	0.47	0.17	0.86
	≥3	185/234	79.1 (73.3-84.1)	Ref.			
**Milking Frequency**	1	122/147	82.9 (75.9-88.7)	0.08	0.47	0.17	0.86
	2	189/238	79.4 (73.7-84.4)	Ref.			
**Previous history of Mastitis**	Yes	161/195	82.6 (76.5-87.6)	0.08	0.47	0.17	0.86
	No	150/190	78.9 (72.5-84.5)	Ref.			
**Body condition score**	≥3	173/213	81.2 (75.3-86.2)	0.49	0.71	0.69	0.49
	2	138/172	80.2 (73.5-85.9)	Ref.			
**Rearing system**	Intensive	109/134	81.3 (73.7-87.6)	0.13	0.36	0.35	0.72
	Semi intensive	58/70	82.9 (71.9-90.8)	0.11	0.35	0.29	0.77
	Household	144/181	79.6 (72.9-85.2)	Ref.			
**Floor type**	Muddy	195/239	81.6 (76.1-86.3)	0.34	0.61	0.55	0.58
	Concrete	35/42	83.3 (68.6-93.0)	0.11	0.36	0.30	0.77
	Sand	81/104	77.9 (68.7-85.4)	Ref.			
**Udder shape**	Cup	57/71	80.3 (69.1-88.8)	0.11	0.36	0.30	0.77
	Bowl	52/62	83.9 (72.3-91.9)	0.13	0.36	0.35	0.72
	Pendulous	192/252	76.2 (70.4-81.3)	Ref.			
**Teat shape**	Large funnel	191/241	79.3 (73.6-84.2)	0.34	0.61	0.55	0.58
	Rounded	69/79	87.3 (77.9-93.8)	0.13	0.36	0.35	0.72
	Flat	51/65	78.5 (66.5-87.7)	Ref.			
**Stocking density**	Medium	128/154	83.1 (76.3-88.7)	0.13	0.36	0.35	0.72
	Proper	20/23	86.9 (66.4-97.2)	0.11	0.35	0.30	0.76
	High	163/208	78.4 (72.1-83.8)	Ref.			

x: number of positive isolates, N: number of samples tested, CI: Confidence interval, Ref.: Reference category; OR: Odds ratio

The multivariate logistic regression analysis did not identify any statistically significant associations between the examined variables and the prevalence of the outcome. For lactation stage, cows in the 3–6 months and >7 months stages showed odds similar to those in the 1–2 months stage, with *p* = 0.98 and *p* = 0.99, respectively. Body condition score had no significant impact, with cows having a score of 3 or more than 3 showing odds comparable to those with a score of 2 (*p* = 0.49). Similarly, rearing system and stocking density did not significantly affect the outcome, with (*p* > 0.70) for all comparisons. These results indicated that none of the studied factors were strongly associated with the prevalence of the outcome.

### Prevalence of *Staphylococcus* and *Streptococcus* species

As shown in Table 4, the MWST identified 1,048 quarter milk samples (68.1%) as positive, a finding supported by the CMT with a comparable positivity rate of 67.9%. Among the total samples, 51.2% (789/1540) of *Staphylococcus* spp. and 27.5% (424/1540) of *Streptococcus* spp. isolated based on culture and biochemical test. Among culture-positive *Staphylococcus* samples, PCR analysis confirmed 627 isolates, with 72.7% (456/627) identified as *S. aureus* and 27.3% (171/627) as Non-aureus *Staphylococcus* (NAS). For *Streptococcus* spp., PCR detected 248 isolates, predominantly *Streptococcus uberis* (32.3%), followed by *Streptococcus dysgalactiae* (14.9%) and *Streptococcus agalactiae* (11.7%). Multiple *Streptococcus* spp. co-existed in 11.7% (29/248) of cases, particularly affecting right quarters, while left quarters had higher incidences of NAS and various *Streptococcus* spp. (*Streptococcus agalactiae, Streptococcus dysgalactiae, and Streptococcus uberis*) infections.

**Table 4:** Descriptive analysis especially Prevalence (Expressed as percent) of SCM pathogens among various diagnostic approaches isolated from Buffalo milk samples (N = 1540) in Sylhet, Bangladesh.

Quarter	Screening Test (n, %)				PCR (n, %)							mPCR
	CMT	MWST	C & B for *Staphylococcus*	C & B for *Streptococcus*	*Staphylococcus* spp.	*S. aureus*	NAS	*Streptococcus* spp.	*S. agalactiae*	*S. dysgalactiae*	*S. uberis*	CoES
LF	270 (70.1)	272 (70.6)	197 (51.2)	125 (32.5)	159 (41.3)	97 (61.0)	62 (39.0)	80 (20.8)	17 (21.3)	10 (12.5)	33 (41.3)	10 (12.5)
LR	260 (67.5)	260 (67.5)	194 (50.4)	101 (26.2)	158 (41.0)	107 (67.7)	51 (32.3)	57 (14.8)	8 (14.0)	13 (22.8)	12 (21.1)	1 (1.8)
RF	254 (66.0)	254 (66.0)	197 (51.2)	98 (25.5)	161 (42.3)	136 (84.5)	25 (15.5)	59 (15.3)	2 (3.4)	11 (18.6)	23 (39.0)	2 (3.4)
RR	262 (68.1)	262 (68.1)	201 (52.2)	100 (26.0)	149 (38.7)	116 (77.9)	33 (22.1)	52 (13.5)	2 (3.8)	3 (5.8)	12 (23.1)	16 (30.8)
**Total**	**1046 (67.9)**	**1048 (68.1)**	**789 (51.2)**	**424 (27.5)**	**627 (40.7)**	**456 (72.7)**	**171 (27.3)**	**248 (16.1)**	**29 (11.7)**	**37 (14.9)**	**80 (32.3)**	**29 (11.7)**

n: No. of positive isolates, LF: Left front, LR: Left rear, RF: Right front, RR: Right rear, C & B: Culture and Biochemical test, NAS: Non-*aureus staphylococci*; CoES: Co-existence for *Streptococcus* spp., mPCR: Multiplex PCR, (): parenthesis indicates %

### Test efficacy: MWST

The MWST demonstrated excellent diagnostic efficacy in identifying SCM cases. With a sensitivity of 99.8% (95% CI: 99.3–99.9) and specificity of 99.2% (95% CI: 97.9–99.8), the test effectively identified true positive and true negative cases. Overall, the MWST achieved an accuracy of 99.6% (95% CI: 99.2–99.9), underscoring its utility as a reliable diagnostic tool for detecting SCM in buffaloes (Table 5, S3 Table).

**Table 5:** Descriptive analysis of the diagnostic performance of the Modified Whiteside Test (MWST) as a screening test for SCM. The table presents the sensitivity, specificity, predictive values, likelihood ratios, and overall accuracy of MWST, along with their 95% confidence intervals (CI).

Parameters	MWST (%)	(95% CI)
Sensitivity	99.8	99.3-99.9
Specificity	99.2	97.9-99.8
Positive Predictive Value (PPV)	99.6	99.0-99.9
Negative Predictive Value (NPV)	99.6	98.5-99.9
Positive Likelihood Ratio (LR^+^)	124.75	N/A
Negative Likelihood Ratio (LR^-^)	0.002	N/A
Accuracy	99.6	99.2-99.9

MWST: Modified Whiteside Test, CI: Confidence Interval, N/A: Not Applicable.

### MRSA and MSSA

**Fig 2:**
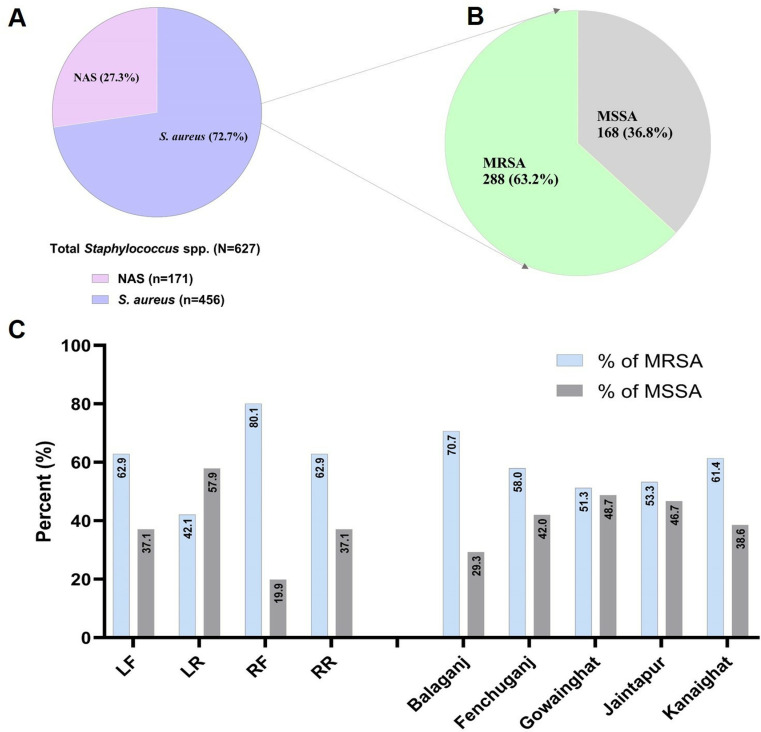
The overall quarter and habitat wise distribution of *Staphylococcus* spp. 2A. Prevalence of *S. aureus* and non-aureus staphylococci, 2B: Percent of MRSA and MSSA, 2C: Quarter and habitat wise distribution of MRSA and MSSA.

The distribution of *Staphylococcus* species among 627 isolates is illustrated, with *S. aureus* constituting 72.7% (n = 456) and NAS making up 27.3% (n = 171). The focus on the *S. aureus* isolates revealed that 63.2% (n = 288) were identified as MRSA, while 36.8% (n = 168) were classified as methicillin-susceptible *S. aureus* (MSSA) as shown in Fig 2. The data demonstrated that MRSA prevalence consistently exceeded that of MSSA across regions, with the highest MRSA rate recorded in Balaganj at 70.7% and the lowest in Gowainghat at 51.3%. MSSA prevalence was found to be highest in Gowainghat at 48.7% and lowest in Balaganj at 29.3%. Anatomically, MRSA was most prevalent in the right front quarter (80.1%), while MSSA peaked in the left rear quarter, highlighting significant geographical and anatomical variability in their distribution.

### Determination of antimicrobial resistant genes

**Fig 3:**
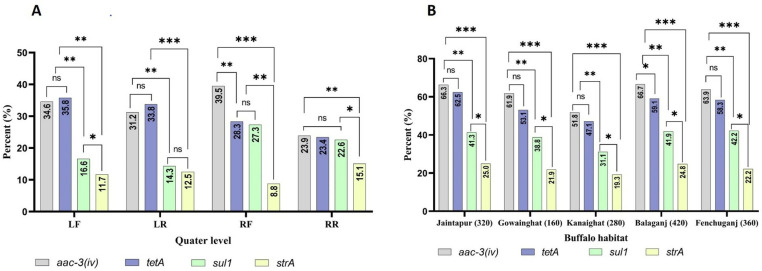
Quarter (Fig 3A) and habitat (Fig 3B) wise determination of antibiotic resistant genes (ARGs), *p* < 0.05, *p* < 0.01, *p* < 0.001.

The prevalence of four ARGs *aac-3(iv), tetA, sul1,* and *strA* across buffalo udder quarters are depicted in Fig 3. In the Left Front (LF) quarter, *aac-3(iv)* was present in 34.6% of samples, and *tetA* in 35.8%, with no significant difference. The *sul1* gene (16.6%) was more common than the *strA* gene (11.7%), with *strA* being significantly lower than *sul1* (*p* < 0.05). In the Left Rear (LR) quarter, *aac-3(iv)* was found in 31.2% of samples, with *tetA* at 33.8%. The *sul1* was present in 14.3%, while *strA* occurred in 12.5%, with no significant difference between them. In the Right Front (RF) quarter, *aac-3(iv)* had the highest prevalence (39.5%), significantly greater than *tetA* (28.3%) *(p* < 0.01). The *sul1* (27.3%) was comparable to that of *tetA* (28.3%)*,* while *strA* (8.8%) was significantly lower than both *tetA* and *sul1 (p* < 0.01). In the Right Rear (RR) quarter, *aac-3(iv)* and *tetA* had similar rates of 23.9% and 23.4%, respectively, while *sul1* was at 22.6%. The *strA* (15.1%) was significantly lower compared to *sul1* (*p* < 0.05).

In Jaintapur, *aac-3(iv)* was detected in 66.3% of samples, significantly higher than *sul1* (41.3%) and *strA* (25.0%) (*p* < 0.001). A similar trend was observed in Gowainghat, where *aac-3(iv)* was found in 61.9% samples and *tetA* in 53.1%, both significantly higher than *sul1* (38.8%) and *strA* (21.9%). In Kanaighat, *aac-3(iv)* (51.8%) and *tetA* (47.1%) were more prevalent than *sul1* (31.1%) and *strA* (19.3%). Balaganj had the highest prevalence of *aac-3(iv)* at (66.7%), while Fenchuganj showed similar trends, with *aac-3(iv)* at 63.9% and *tetA* at 58.3%. Across all habitats, *sul1* and *strA* were consistently less prevalent, with significant differences observed between *aac-3(iv)* and *strA* (*p* < 0.001) and between *sul1* and *strA* (*p* < 0.05).

### Antimicrobial susceptibility profiling

The antibiotic susceptibility profiles of 627 *Staphylococcus* spp. and 248 *Streptococcus* spp. isolates are detailed in Table 6. The *Staphylococcus* spp. displayed significant susceptibility to trimethoprim-sulfamethoxazole (87.4%), tetracycline (80.9%), and streptomycin (76.7%), while substantial resistance was noted against ampicillin (88.8%) and nalidixic acid (68.1%). Similarly, *Streptococcus* spp. demonstrated pronounced sensitivity to tetracycline (83.1%), sulfamethoxazole-trimethoprim (81.9%), streptomycin (81.1%), and cefoxitin (81.0%), yet considerable resistance was observed against ampicillin (87.1%) and nalidixic acid (62.1%).

**Table 6:** Descriptive analysis of the antimicrobial resistance profile of *Staphylococcus* spp. and *Streptococcus* spp. isolated from SCM cases. The table presents the distribution of resistant (R), intermediate (I), and sensitive (S) isolates, along with the interpretation criteria and specific disk potency (µg) for each antibiotic tested.

Organism	Antimicrobial class	Antibiotics	Disc potency (ug)	Interpretation			Results (%, n)		
				S	I	R	S	I	R
*Staphylococcus* spp.	Penicillins	Ampicillin (AMP)	10	≥29	---	≤28	11.2 (70)	---	88.8 (557)
		Amoxicillin/ Clavulanic acid (AMC)	20/10	≥18	14-17	≤13	45.8 (287)	12.3 (77)	41.9 (263)
	Cephems including Cephalosporin	Cefoxitin (FOX)	30	≥22	---	≤21	57.9 (363)	4.2 (26)	37.9 (238)
		Ceftriaxone (CRO)	30	≥23	20-22	≤19	50.8 (126)	2.4 (6)	46.8 (116)
	Aminoglycosides	Gentamicin (CN)	10	≥15	13-14	≤12	44.2 (277)	3.8 (24)	52.0 (326)
		Amikacin (AK)	30	≥20	17-19	≤16	30.8 (193)	20.2 (127)	49.0 (307)
		Streptomycin (STR)	10	≥15	12-14	≤11	76.7 (481)	2.4 (15)	20.9 (131)
	Tetracyclines	Tetracycline (TET)	30	≥19	15-18	≤14	80.9 (507)	5.2 (33)	13.9 (87)
	Macrolides	Azithromycin (AZM)	15	≥18	14-17	≤13	64.1 (402)	18.8 (118)	17.1 (107)
	Quinolones	Ciprofloxacin (CIP)	5	≥21	16-20	≤15	64.3 (403)	33.0 (207)	2.7 (17)
		Nalidixic acid (NA)	30	≥19	14-18	≤13	24.1 (151)	7.8 (49)	68.1 (427)
	Folate pathway antagonists	Trimethoprim- Sulfamethoxazole (SXT)	1.25/23.75	≥16	11-15	≤10	87.4 (548)	6.5 (41)	6.1 (38)
	Phenicols	Chloramphenicol (C)	30	≥18	13-17	≤12	69.9 (438)	8.1 (51)	22.0 (138)
*Streptococcus* spp.	Penicillins	Ampicillin (AMP)	10	≥24	----	≤23	12.9 (32)	--	87.1 (216)
		Amoxicillin/ Clavulanic acid (AMC)	20/10	≥18	14-17	≤13	59.3 (147)	8.9 (22)	31.9 (79)
	Cephems including Cephalosporin	Cefoxitin (FOX)	30	≥22	---	≤21	81.0 (201)	5.2 (13)	13.7 (34)
		Ceftriaxone (CRO)	30	≥24	---	≤23	57.3 (142)		42.7 (106)
	Aminoglycosides	Gentamicin (CN)	10	≥15	13-14	≤12	56.9 (141)	27.0 (67)	16.1 (40)
		Amikacin (AK)	30	≥20	17-19	≤16	41.9 (104)	5.3 (13)	52.8 (131)
		Streptomycin (STR)	10	≥15	12-14	≤11	81.1 (201)	4.0 (10)	14.9 (37)
	Tetracyclines	Tetracycline (TET)	30	≥23	19-22	≤18	83.1 (206)	---	16.9 (42)
	Macrolides	Azithromycin (AZM)	15	≥18	14-17	≤13	42.7 (106)	6.5 (16)	50.8 (126)
	Quinolones	Ciprofloxacin (CIP)	5	≥21	16-20	≤15	20.2 (50)	41.9 (104)	37.9 (94)
		Nalidixic acid (NA)	30	≥19	14-18	≤13	31.5 (78)	6.4 (16)	62.1 (154)
	Folate pathway antagonists	Trimethoprim- Sulfamethoxazole (SXT)	1.25/23.75	≥16	11-15	≤10	81.9 (203)	4.8 (12)	13.3 (33)
	Phenicols	Chloramphenicol (C)	30	≥21	18-20	≤17	60.9 (151)	1.2 (3)	37.9 (94)

S: Sensitive, I: Intermediate, R: Resistant, n: number of isolates

### MAR index and MDR, XDR patterns

A total of 627 *Staphylococcus* spp. and 248 *Streptococcus* spp. isolates were analyzed for MDR and Multiple Antimicrobial Resistance Index (MARI). In *Staphylococcus* spp., 76.4% (479/627) were considered as MDR, among the MDR species 10.37% (65/627) potentially exhibiting possible XDR, and an average MARI of 0.43. *Streptococcus* spp. showed 67.3% (167/248) MDR, with 10.48% (26/248) possible XDR, and an average MARI of 0.47. The highest MARI recorded for *Staphylococcu*s spp. was 0.92, indicating resistance to 12 out of 13 antibiotics tested. In contrast, a MAR index of 0.85 was observed for *Streptococcus* spp. The dominant resistance genes in both species included *aac-3(iv), tetA*, and *sul1* (Table 7).

**Table 7:** The occurrence of MDR patterns and MAR index among the *Staphylococcus* and *Streptococcus* spp. positive isolates.

Organisms	No. of isolates	%	Phenotypic pattern	Resistance type	Resistant Antimicrobials (Class)	ARGs	MARI
*Staphylococcus* spp.	8	1.3	AMP-AMC-FOX-CRO-CN-AK-TET-AZM-STR-NA-SXT-C	Possible XDR	12 (8)	*aac-3 (iv), tetA, sul1, strA*	0.92
	11	1.8	AMP-AMC-FOX-CRO-CN-AK-TET-AZM-STR-CIP-SXT-C	Possible XDR	12 (8)	*aac-3 (iv), tetA, sul1, strA*	0.92
	35	5.6	AMP-FOX-CN-AK-AZM-NA	MDR	6 (5)	*aac-3 (iv), tetA, strA*	0.46
	15	2.4	AMP-AMC-FOX-CRO-CN-TET-NA-SXT-C	Possible XDR	9 (6)	*aac-3 (iv), tetA, sul1, strA*	0.69
	31	4.9	AMP-AMC-FOX-CN-AK-STR-AZM-C-NA	Possible XDR	9 (7)	*aac-3 (iv), tetA, sul1, strA*	0.69
	83	13.2	AMP-FOX-AK-NA-CN	MDR	5 (4)	*aac-3 (iv), tetA, sul1*	0.38
	71	11.3	AMP-AMC-CN-STR-C	MDR	5 (3)	*tetA, sul1, strA*	0.38
	49	7.8	AMP-FOX-CN-AK-NA	MDR	5 (4)	*aac-3 (iv), tetA*	0.38
	23	3.7	AMP-AMC-AK-STR-NA	MDR	5 (3)	*aac-3 (iv), tetA, strA*	0.38
	17	2.7	AMP-AMC-FOX-CN-AK-AZM-NA	MDR	7 (5)	*aac-3 (iv)*	0.54
	12	1.9	AMP-AMC-AK-SXT	MDR	4 (3)	*sul1*	0.31
	52	8.3	AMP-CN-AK-NA	MDR	4 (3)	*aac-3 (iv), tetA*	0.31
	43	6.9	AMP-CN-AK-NA-C	MDR	5 (4)	*aac-3 (iv), tetA*	0.38
	22	3.5	AMP-AMC-FOX-AK-NA-STR	MDR	6 (4)	*strA*	0.46
	7	1.1	AMP-SXT-C	MDR	3 (3)	*aac-3 (iv), tetA*	0.23
	**Total** **479**	**76.4**					
*Streptococcus* spp.	12	4.8	AMP-AMC-FOX-CRO-AK-TET-AZM-CIP-NA-C	Possible XDR	10 (7)	*aac-3 (iv), tetA, strA*	0.77
	14	5.6	AMP-AMC-CRO-AK-CN-STR-AZM-CIP-NA-SXT-C	Possible XDR	11 (7)	*aac-3 (iv), tetA, sul1, strA*	0.85
	12	4.8	AMP-AMC-CRO-AK-STR-AZM-NA-C	MDR	8 (6)	*aac-3 (iv), sul1, strA*	0.62
	8	3.2	AMP-AMC-CRO-AK-AZM-CIP-NA-SXT	MDR	8 (6)	*tetA, sul1, strA*	0.62
	8	3.2	AMP-CRO-AK-AZM-NA-C	MDR	6 (6)	*aac-3 (iv), strA*	0.46
	17	6.9	AMP-AK-AZM-CIP-NA-C	MDR	6 (6)	*aac-3 (iv), tetA, strA*	0.46
	23	9.3	AMP-AK-TET-CIP-NA	MDR	5 (5)	*aac-3 (iv), tetA, sul1*	0.38
	22	8.9	AMP-AK-NA-C	MDR	4 (4)	*strA*	0.31
	13	5.2	AMP-AMC-CRO-AZM-NA	MDR	5 (4)	*aac-3 (iv), tetA*	0.38
	9	3.6	AMP-NA-C-SXT	MDR	4 (4)	*aac-3 (iv), sul1*	0.31
	17	6.9	AMC-CRO-CN-STR-AZM-NA	MDR	6 (5)	*aac-3 (iv), strA*	0.46
	5	2.0	AMP-NA-AZM	MDR	3 (3)		0.23
	7	2.8	AMP-CRO-AK-AZM-C	MDR	5 (5)	*aac-3 (iv), tetA*	0.38
	**Total** **167**	**67.3**					

ARGs: Antibiotic resistant genes, MARI: Multiple antibiotic resistant index

## Discussion

This study comprehensively investigated SCM in buffaloes in the Sylhet district of Bangladesh, focusing on its prevalence and major bacterial pathogens, particularly *Staphylococcus* and *Streptococcus* species. The investigation revealed a prevalence of SCM at 67.9% at the quarter level and 80.8% at the animal level. The animal-level prevalence of SCM in this study closely parallels findings from previous research conducted in Bangladesh and Pakistan (81.6%, 75%, 75.31%) [11,50,51]. However, these rates are significantly higher than those observed in other studies from Bangladesh (47%, 52%, 53.85%), India (26.20%), Pakistan (51%), and the Philippines (42.76%) [9,37,52–56]. Additionally, they exceed the 64.92% prevalence reported in another investigation of bovine SCM in the Sylhet district [37].The quarter-level prevalence of SCM in this investigation closely corresponds with the findings from India (45.8–61.6%) [42] and Pakistan (68.63%) [50]. Conversely, studies conducted in Bangladesh reported considerably lower rates (27%−42.5%) than the findings of the current study [11,20,29]. Additionally, the prevalence is markedly higher than the 19.3% noted in Chitwan, Nepal, underscoring the regional variability in SCM prevalence [11,16,52,56]. The higher animal-level prevalence of SCM compared to the quarter level is due to a single SCM-positive quarter designating the entire buffalo as SCM-positive [52]. SCM prevalence varies by country and is influenced by factors like hygiene practices, milking techniques, lactation stage, and genetics. The observed high prevalence in Gowainghat likely reflects localized conditions in the selected high-pathogen-burden clusters rather than the entire region. Contributing factors include the area’s humid environment, poor management practices, and limited sample sizes. A larger, more representative sample from diverse clusters would provide a more accurate depiction of SCM prevalence in the region. These findings are notably higher than previous studies in Bangladesh [7,9,57]. The variability in SCM prevalence may result from differences in geography, climate, housing systems, milking practices, udder cleanliness, hygiene protocols, and biosecurity awareness among livestock owners [56,58].

The present study found that, buffaloes in early (1–2 months) and late (>7 months) lactation stages were more susceptible to SCM consistent with findings from some studies in Bangladesh and India but conflicting with others in India and Nepal that reported higher SCM prevalence during mid-lactation [59–62]. Regarding parity, early parity buffaloes exhibited higher SCM rates, with prevalence decreasing as parity increased, aligning with a study in Bangladesh but differing from research in Bangladesh, India, and Egypt [56,59,60,63]. Additionally, the study noted that increased milking frequency was associated with lower SCM prevalence. This finding is corroborated by research from Nepal, which indicated that buffaloes milked three times daily had reduced somatic cell counts (SCC) compared to those milked twice daily [64]. Increased milking frequency helps reduce SCM by lowering bacterial load and residual milk volume, which might otherwise promote bacterial growth [64]. A history of mastitis also elevated SCM prevalence, aligning with findings from coastal region of Bangladesh, where buffaloes with peri-parturient diseases like clinical mastitis showed higher SCM rates [59]. This could be due to udder tissue damage, altered immunity, persistent pathogens, and increased SCC. Interestingly, the study showed that higher BCS is associated with higher SCM prevalence, contrasting with other studies suggesting that lower BCS is associated with higher SCC and SCM [56,65]. Poor body condition in buffaloes may lead to increased stress and weakened immune responses, making them more susceptible to bacterial colonization in the mammary gland, especially under suboptimal management and hygiene conditions [2]. In addition, SCM was most prevalent (82.9%) in buffaloes reared under semi-intensive systems, contrary to findings in other studies that linked intensive rearing with higher SCM rates due to stress from limited grazing and wallowing which can compromise the immunity of the animals [52,66]. Buffaloes raised on muddy floors showed increased SCM prevalence, as moist, dirty environments promote pathogen growth and their transmission, aligning with studies from Bangladesh, Pakistan, and Kenya [67–69]. Moreover, buffaloes with longer, funnel-shaped teats and pendulous udders were more prone to SCM, as these traits increase susceptibility to injury and environmental contamination, consistent with findings from Bangladesh and India [52,56,62,70–72]. Higher stocking densities also elevated SCM risk, corroborating similar studies in Bangladesh and South Ethiopia [52,73]. However, the prevalence of different variable categories was relatively similar, and our study did not identify any biologically plausible risk factors for the occurrence of SCM in buffalo. Therefore, it can be suggested that the occurrence of SCM is influenced by a combination of factors rather than a single individual factor.

The MWST is also considered as the most promising diagnostic test for initial screening of SCM. It is cost effective than CMT. Overall, the MWST achieved an accuracy of 99.6%, underscoring its utility as a reliable diagnostic tool for detecting SCM in buffaloes.

Culture and biochemical analysis showed 51.2% of isolates were *Staphylococcus* spp. and 27.5% were *Streptococcus* spp., contrasting with studies from India and Bangladesh that reported 42%−71.28% for *Staphylococcus* and 14%−17.95% for *Streptococcus* species [20,42]. The PCR confirmed *S. aureus* as the predominant species (72.7%), consistent with research from Bangladesh and Pakistan but diverging from recent studies in Bangladesh [20,56,59,68,70,74]. Moreover, *Streptococcus uberis* (32.3%) was the most common *Streptococcus* spp.*,* followed by *Streptococcus dysgalactiae* (14.9%), aligning with studies from Bangladesh and Korea but contradicted with the findings from India, where *Streptococcus dysgalactiae* was predominant [23,75,76]. SCM prevalence varies with diagnostic methods. Rapid tests like MWST and CMT offer quick results but less precision, while PCR, the most sensitive method, detects low bacterial DNA, increasing SCM prevalence reports [11]. In our study, *S. aureus* predominantly affected right-sided quarters, consistent with findings in Pakistan but differing from India and Bangladesh, where left rear quarters were more affected [55,70,77]. NAS and *Streptococcus* spp. were more prevalent in left-sided quarters, likely due to less effective milking and increased pathogen exposure. Right-sided quarters, producing more milk, fostering co-existence of multiple *Streptococcus* spp., while left-sided quarters were dominated by single *Streptococcus* spp. due to lower milk production and stronger immunity [78].

Among the 627 *Staphylococcus* isolates, *S. aureus* predominated, representing 72.7% (456 isolates), with 63.2% identified as MRSA and 36.8% as MSSA. This prevalence of MRSA aligns with studies conducted in Egypt but contrasts with findings from Bangladesh, Pakistan, and Nepal [15,20,79–81]. The elevated prevalence of MRSA highlights a significant public health issue, reflecting widespread antibiotic resistance. MRSA was consistently more prevalent than MSSA across all regions studied. Furthermore, the study found higher MRSA prevalence in the RF and RR quarters, while MSSA was more common in the LR quarter, suggesting spatial variation in infections. This distribution may be influenced by regional disparities in antimicrobial use, infection control strategies, and milking practices. Increased environmental pathogen exposure or mechanical stress in the right quarters may favor MRSA, while MSSA persists in the left due to varying ecological pressures and resistance dynamic.

Furthermore, a comparable distribution of ARGs has been noted in bovine SCM, with *tetA* frequently detected [82]. But in our study, *tetA* prevalence ranged from 23.4% to 35.8%, highest in the LF quarter (35.8%) and lowest in the RR quarter (23.4%). Interestingly, the RF quarter exhibited a significantly higher prevalence of *aac-3(iv)* (39.5%) compared to *tetA* (28.3%) (*p* < 0.01), likely due to species-specific usage or genetic variations in ARGs dissemination between buffaloes and cattle. The *sul1* gene, associated with sulfonamide resistance, varied from 14.3% to 27.3%, with the RF quarter showing the highest prevalence, contrasting with findings from Egypt where *sul1* was 100% [83]. This lower prevalence may indicate regional differences in antibiotic usage. The *strA* gene, conferring streptomycin resistance, was the least prevalent, ranging from 8.8% to 15.1%, possibly reflecting reduced streptomycin usage or geographic differences in aminoglycoside resistance. In addition, the widespread prevalence of *aac-3(iv)* and *tetA* across habitats suggests environmental factors, genetic predispositions, and management practices influence ARG distribution [84]. Although *sul1* and *strA* were less common, their presence remains significant, highlighting a complex interplay of factors affecting ARG prevalence. Statistically significant differences between ARGs emphasize the need to understand ecological and human factors to develop targeted strategies against antibiotic resistance.

A higher susceptibility (87.4%) of *Staphylococcus* spp. isolates was observed against trimethoprim-sulfamethoxazole, aligning with reports of its high efficacy and minimal resistance (0.96%), indicating its continued reliability as a treatment option [85]. Tetracycline also exhibited elevated sensitivity in this study, which contrasts with findings from South Africa, where tetracycline sensitivity was reported at 56.7% [86]. In the context of Bangladesh, both tetracycline and gentamicin are commonly used for treating SCM. In our study, we found that both antibiotics appeared phenotypically sensitive but showed genotypic resistance. The presence of *tetA* does not always result in phenotypic resistance. Resistance genes can exist in a “silent” state, where they are either transcriptionally inactive or expressed at levels too low to confer detectable resistance under standard antimicrobial susceptibility testing (AST) conditions [87,88]. The expression of *tetA* is often regulated by inducible promoters, such as those triggered by the presence of tetracycline or related compounds. In the absence of such inducers during AST, the gene may not be actively transcribed, resulting in a susceptible phenotype [89]. Environmental conditions, stress signals, or co-resistance gene activation may be required for full expression.

In contrast, ampicillin and nalidixic acid showed highest resistance to *Staphylococcus* spp., consistent with findings from West Java [90]. Tetracycline and trimethoprim-sulfamethoxazole exhibited strong sensitivity by inhibiting protein and folic acid synthesis, respectively, while resistance to ampicillin and nalidixic acid arises from beta-lactamase production and DNA gyrase mutations [91]. Likewise, tetracycline, trimethoprim-sulfamethoxazole, streptomycin, and cefoxitin demonstrated remarkable sensitivity (>80%) to *Streptococcus* spp. However, previous research from France and China revealed elevated resistance to tetracycline, which contrasts with our findings [92,93]. In contrast, trimethoprim-sulfamethoxazole exhibited 100% sensitivity in the USA, which closely aligns with our results [94]. Additionally, lower resistance to streptomycin (11–16%) in France and cefoxitin further corroborate the outcomes observed in this study [92,95]. On the contrary, ampicillin and nalidixic acid exhibited highest resistance to *Streptococcus* spp. in this study. A Swiss study reported over 90% susceptibility to ampicillin, conflicting with our findings, while nalidixic acid showing 100% resistance to *Streptococcus* spp. [92,96].

Furthermore, this study reported a 76.4% MDR rate for *Staphylococcus* spp., aligning with previous research showing 89.2% MDR in *S. aureus* from clinical samples. However, it contrasts with findings from Shanghai, China, where only 39.4% MDR was found in animal-based food samples [97,98]. The XDR prevalence here also exceeds other reports, which detected just 1.58% [97]. It can be attributed to the organism’s ability to acquire resistance genes through horizontal gene transfer and its adaptability in diverse environments, particularly in healthcare settings. Additionally, factors such as antibiotic misuse and inadequate infection control measures have been implicated in facilitating the emergence and spread of XDR *S. aureus* strains [99]. MARI values for *Staphylococcus* spp. were slightly higher than previous findings, where MARI ranged from 0.2 to 0.6. Variations in MDR and MARI values may be due to differences in antibiotic use, bacterial sources, testing methodologies, geographic and temporal factors, and diversity of the study populations, highlighting the complexity of resistance patterns across studies [100]. In contrast, The MDR rate for *Streptococcus* spp., (67.3%) significantly higher than rates reported in Bangladesh (30.8%) and Ethiopia (46.1%), while the XDR rate (10.4%) was much higher than 1.2% reported in South Korea [101–103]. These disparities in MDR and XDR prevalence are likely due to differences in antibiotic prescribing practices, healthcare infrastructure, and surveillance systems. Countries with stringent antibiotic stewardship report lower resistance rates, whereas regions with higher antibiotic misuse and inadequate healthcare access face increased MDR and XDR prevalence. Additionally, Socio-economic factors, population density, and infection prevalence further influence these patterns. The MARI value for *Streptococcus* spp. is also elevated, highlighting local environmental and clinical factors affecting antibiotic resistance profiles [104,105]. The lack of significant associations found in the multivariate logistic regression analysis suggests that the factors considered, such as lactation stage, body condition score, rearing system, and stocking density etc., may not be the primary determinants of SCM in buffaloes. These findings highlight the need for further research to explore additional factors, such as management practices, environmental conditions, or genetic predispositions, that could play a more substantial role in SCM prevalence.

This study limits the sample size for certain groups which was relatively small, which may affect the generalizability of the findings of that particular group. Additionally, variations in environmental factors across different animal cluster may have influenced the results, although efforts were made to standardize the conditions. The use of PCR for pathogen identification, while reliable, may not capture all potential microbial species associated with subclinical mastitis.

## Conclusion

This study explored the high prevalence of MDR and possible XDR *Staphylococcus* and *Streptococcus* species in raw buffalo milk from SCM cases in Bangladesh. With an SCM prevalence of 67.9% at the quarter level and 80.8% at the animal level, the findings highlighted the urgent need for enhanced monitoring and management strategies within the dairy industry. Notably, 63.2% of *S. aureus* isolates were methicillin-resistant, and a high prevalence of ARGs such as *aac-3(iv)* and *tetA* was identified. The observed high resistance to key antibiotics, particularly ampicillin and nalidixic acid, alongside significant susceptibility to tetracycline and trimethoprim-sulfamethoxazole, further emphasizes public health risks associated with these resistant pathogens. The recorded MAR indices for both *Staphylococcus* spp. (0.92) and *Streptococcus* spp. (0.85) indicated widespread MDR. This study highlighted the pressing challenge of managing SCM in buffaloes amidst rising antimicrobial resistance, calling for targeted interventions to mitigate risks to animal health, milk production, and public health.

## Supporting information

S1 Table Reaction mixture and thermal cycling condition for molecular detection of different organisms. (DOCX)

S2 Table CMT grading of SCM positive samples (n = 1046). (DOCX)

S3 Table Cross tabulation of CMT (Most accepted screening test) and MWST (Screening test) for diagnosis of SCM in Buffaloes. (DOCX)
