# Supplementary material for: Detection and prevalence of antimicrobial resistance genes in multidrug-resistant and extensively drug-resistant Staphylococcus and Streptococcus species isolated from raw buffalo milk in subclinical mastitis
Source: PLoS One. 2025 Jun 17;20(6):e0324920. doi: 10.1371/journal.pone.0324920 (PMC12173402; doi:10.1371/journal.pone.0324920)
Supplement: S1 Table — (DOCX) [file pone.0324920.s001.docx]

**S1 Table: Reaction mixture and thermal cycling condition for molecular detection of different organisms**

| **Organism** | **Thermal cycle** | **Reaction mixture (25 μl)** |
| --- | --- | --- |
| Genus Streptococcus | - **I**nitial denaturation at 94°C for 5 minutes - 35 cycles of denaturation at 95°C for 60 seconds, annealing for 30 seconds at 50°C, and elongation at 72°C for 2 minutes - Final elongation at 72°C for 10 minutes | - 2x master mix (Add Bio Inc, South Korea) at 12.5 μl - 1 μl per primer (forward and reverse) at a concentration of 10 pmol/L - Template DNA 5 μl - Nuclease-free water 5.5 μl |
| *Staphylococcus aureus* | - Initial denaturation at 94°C for 5 minutes - 35 cycles of denaturation at 94°C for 60 seconds, annealing for 30 seconds at 55°C, and elongation at 72°C for 1.5 minutes - Final elongation at 72°C for 3.5 minutes | - 2x master mix (Add Bio Inc, South Korea) at 12.5 μl - 1 μl per primer (forward and reverse) at a concentration of 10 pmol/L - Template DNA 5 μl - Nuclease-free water 5.5 μl |
| Methicillin resistant *Staphylococcus aureus*  (MRSA) | - Initial denaturation for 5 minutes at 94°C - 30 cycles of denaturation for 60 seconds at 94°C, annealing for 45 seconds at 55°C, and elongation at 72°C for 45 seconds - Final elongation at 72°C for 5 minutes | - 2x master mix (Add Bio Inc, South Korea) at 12.5 μl - 1 μl per primer (forward and reverse) at a concentration of 10 pmol/L - Template DNA 5 μl - Nuclease-free water 5.5 μl |
| *Streptococcus agalactiae, Streptococcus dysgalactiae, Streptococcus uberis* | - Initial denaturation for 5 minutes at 94°C - 35 cycles of denaturation for 60 seconds at 95°C, annealing for 30 seconds at 57°C, and extension for 2 minutes at 72°C - Final extension at 72°C for 10 minutes | - 2x master mix (Add Bio Inc, South Korea) at 12.5 μl - 0.5 μl per primer (forward and reverse) at a concentration of 10 pmol/L - Template DNA 5 μl - Nuclease-free water 4.5 μl |
| Gentamycin resistant gene *AAC (3)-iv* | - Initial denaturation for 10 minutes at 94°C - 35 cycles of denaturation for 60 seconds at 94°C, annealing for 1 minute at 63°C, extension at 72°C for 1 minute - Final extension at 72°C for 10 minutes | - 2x master mix (Add Bio Inc, South Korea) at 12.5 μl - 1 μl per primer (forward and reverse) at a concentration of 10 pmol/L - Template DNA 5 μl - Nuclease-free water 5.5 μl |
| Sulphonamide resistant gene *Sul1* | - Initial denaturation for 15 minutes at 95°C - 30 cycles of denaturation for 60 seconds at 95°C, annealing for 1 minute at 66°C, extension at 72°C for 1 minute - Final extension at 72°C for 10 minutes | - 2x master mix (Add Bio Inc, South Korea) at 12.5 μl - 1 μl per primer (forward and reverse) at a concentration of 10 pmol/L - Template DNA 5 μl - Nuclease-free water 5.5 μl |
| Tetracycline resistant gene *tetA*, Streptomycin resistant gene *strA* | - Initial denaturation at 94°C for 15 minutes, - 30 cycles of 1 minute at 94°C for denaturation,   1 minute at 63°C for primer annealing, 1minute at 72°C for strand elongation   - Final elongation at 72°C for 10 minutes | - 2x master mix (Add Bio Inc, South Korea) at 12.5 μl - 0.5 μl per primer (forward and reverse) at a concentration of 10 pmol/L - Template DNA 5 μl - Nuclease-free water 5.5 μl |
