# Supplementary material for: Detection and prevalence of antimicrobial resistance genes in multidrug-resistant and extensively drug-resistant Staphylococcus and Streptococcus species isolated from raw buffalo milk in subclinical mastitis
Source: PLoS One. 2025 Jun 17;20(6):e0324920. doi: 10.1371/journal.pone.0324920 (PMC12173402; doi:10.1371/journal.pone.0324920)
Supplement: S2 Table — (DOCX) [file pone.0324920.s002.docx]

**S2 Table:** **CMT grading of SCM positive samples (n=1046)**

| **Grading** | **No of positive samples** | **%; 95% CI** |
| --- | --- | --- |
| Trace | 392 | 37.48 (34.5-40.5) |
| 1+ | 214 | 20.46 (18.1-23.0) |
| 2+ | 293 | 28.01 (25.3-30.8) |
| 3+ | 147 | 14.05 (12.0-16.3) |
