# Supplementary material for: Detection and prevalence of antimicrobial resistance genes in multidrug-resistant and extensively drug-resistant Staphylococcus and Streptococcus species isolated from raw buffalo milk in subclinical mastitis
Source: PLoS One. 2025 Jun 17;20(6):e0324920. doi: 10.1371/journal.pone.0324920 (PMC12173402; doi:10.1371/journal.pone.0324920)
Supplement: S3 Table — (DOCX) [file pone.0324920.s003.docx]

**S3 Table:** **Cross tabulation of CMT (Most accepted screening test) and MWST (Screening test) for diagnosis of SCM in Buffaloes**

| **Test Name** | **CMT (Positive)** | **CMT (Negative)** | **Total** | |
| --- | --- | --- | --- | --- |
| MWST (Positive) | 1044 | 4 | | 1048 |
| MWST (Negative) | 2 | 490 | | 492 |
| Total | 1046 | 494 | | 1540 |
